# Carbapenemase type and mortality in blood-stream infections caused by carbapenemase-producing enterobacterales: a multicenter retrospective cohort study

**DOI:** 10.1007/s15010-025-02584-y

**Published:** 2025-06-16

**Authors:** Yaakov Dickstein, Dafna Yahav, Giusy Tiseo, Cristina Mussini, Erica Franceschini, Antonella Santoro, Galia Rahav, Hila Elinav, Assaf Potruch, Amir Nutman, Mical Paul, Marco Falcone

**Affiliations:** 1 Infectious Diseases Unit, Rambam Health Care Campus, Haifa, Israel; 2 Infectious Diseases Unit, Sheba Medical Center, Derech Sheba 2, Ramat Gan, 52621 Israel; 3 Faculty of medical Health Sciences, Tel-Aviv University, Ramat-Aviv, Tel-Aviv, Israel; 4 Department of Clinical and Experimental Medicine, Azienda Osperdaliero Universitaria Pisana, University of Pisa, Pisa, Italy; 5 Department of Infectious Diseases, Azienda Ospedaliero-Universitaria, Policlinico of Modena, Modena, Italy; 6 Department of Surgical, Medical, Dental, and Morphological Sciences, University of Modena and Reggio Emilia, Infectious Disease Clinic University of Modena and Reggio Emilia AOU Policlinico di Modena, Modena, Italy; 7 Department of Microbiology and Infectious Diseases, Hadassah Medical Center, Jerusalem, Israel; 8 Faculty of Medicine, Hebrew University of Jerusalem, Jerusalem, Israel; 9 Department of Nephrology and Hypertension, Hadassah Medical Center, Jerusalem, Israel; 10 Hospital management, Wolfson Medical Center, Holon, Israel; 11 The Ruth and Bruce Rappaport Faculty of Medicine, Technion Israel Institute of Technology, Haifa, Israel

**Keywords:** Aztreonam, Blood-stream infection, Ceftazidime-avibactam, Colistin, CPE, KPC, NDM

## Abstract

**Background:**

Previous studies analyzing differences in mortality associated with carbapenemase type in patients with a variety of infections caused by carbapenemase-producing *Enterobacterales* (CPE) have produced conflicting results.

**Methods:**

We performed a multinational multicenter retrospective cohort study. Adult patients with blood-stream infections (BSI) caused by CPE between 2015 and 2020 were included. The primary outcome was 14-day mortality; 28-day mortality and microbiological failure were secondary outcomes. Clinical and microbiological data were collected and analyzed using conditional logistic regression.

**Results:**

A total of 360 patients were identified of whom 226 had infections caused by KPC-producing isolates, 109 by NDM-producing isolates and 25 by other carbapenemases. Definitive therapy was colistin-based in 35.1% of patients, ceftazidime/avibactam ± aztreonam (CAZ/AVI ± A) in 28.2% and other in 23.4%. Overall 14-day mortality was 28.1%; carbapenemase type was unassociated with mortality in univariate or multivariate analyses. Antimicrobial therapy was significantly associated with 14-day mortality: patients treated with CAZ/AVI ± A had an adjusted hazard ratio of 0.172 (95% confidence interval 0.063–0.473) for death as compared to patients treated with colistin-based therapy. At 28 days, overall mortality was 35.3%; no association was observed between carbapenemase type and 28-day mortality or microbiological failure.

**Conclusion:**

After controlling for antimicrobial therapy, we did not find evidence of an association between carbapenemase type and mortality. Ceftazidime/avibactam was associated with a greater than 80% reduction in mortality as compared with colistin.

**Supplementary Information:**

The online version contains supplementary material available at 10.1007/s15010-025-02584-y.

## Abstract (plain language summary)

We analyzed differences in mortality associated with different carbapenemase types in patients with infections caused by carbapenemase-producing *Enterobacterales*. No evidence of an association was found, however therapy with ceftazidime/avibactam was associated with a reduction in mortality as compared with colistin.

**Supplementary Information:**

The online version contains supplementary material available at 10.1007/s15010-025-02584-y.

## Background

Blood-stream infections (BSI) due to carbapenemase-producing *Enterobacterales* (CPE) are associated with considerable mortality [1, 2]. Studies comparing mortality in patients colonized with NDM- and KPC-producing CPE have identified higher mortality rates among those colonized by KPC [3, 4]. It is unclear, however, whether these findings are due to confounding or if carbapenemase type is itself associated with virulence. The results of studies analyzing mortality by carbapenemase type in patients with a variety of infections caused by CPE have been conflicting [3, 5]. Studies of patients with bloodstream infections caused by CPE have included mainly KPC-producing isolates and as a result there has been little opportunity to compare the outcomes of patients with differing mechanisms of CPE bacteremia [6–9]. Articles comparing outcomes in patients with bacteremia caused by CPE have produced differing results [2, 10, 11]. In the present study we aimed to assess whether there is a difference in the mortality of bloodstream infections due to CPE producing different types of carbapenemase.

## Methods

We performed a retrospective observational study comparing patients with CPE bacteremia due to different carbapenemases including KPC, NDM and OXA-48. We included patients from seven hospitals in Israel and Italy between the years 2015–2020. Inclusion criteria were age ≥ 18, documented bacteremia caused by carbapanemase-producing *Enterobacterales* and molecular identification of CPE type. Patients not meeting these criteria were excluded. Patients with more than one episode of CPE bacteremia were included for the first episode only.

The primary efficacy outcome was 14-day mortality. Secondary outcomes included 28-day mortality and microbiological failure, defined as recurrence of bacteremia with the same CPE within 28 days. We gathered patient data including demographic information, preexisting medical conditions and information pertaining to the infection from hospital database and electronic medical records. Definitive therapy was defined as the first appropriate antimicrobial with which a patient was treated for ≥ 3 days following culture taken date (CTD). For patients who received combination therapy, a hierarchy was defined in which ceftazidime/avibactam ± aztreonam (CAZ/AVI ± A) was first followed by colistin followed by other appropriate antibiotics (OAA). For example, a patient who received combination therapy with CAZ/AVI ± A and colistin would be defined as receiving definitive therapy with CAZ/AVI ± A while a patient receiving colistin and amikacin would be defined as receiving definitive therapy with colistin. Colistin was administered with a loading dose of 9 M IU followed by 4.5 M IU every 12 h and dose adjustment for renal function was performed according to current guidelines and manufacturer instructions.

We calculated variable medians (inter-quartile range [IQR]) and frequencies as appropriate. Univariate analysis was performed for continuous variables using Student’s *t*-test, Mann-Whitney *U* test or Kruskall-Wallis *H* test and for categorical variables using X^2^ or Fisher’s exact test as appropriate. For analyses of mortality, all variables found to be statistically significant at a level of *p* < 0.1 on univariate analysis and not correlated were included in the multivariable analysis performed with conditional logistic regression. To further control for the effects of confounding, additional analyses were performed separately for patients who received definitive therapy with CAZ/AVI ± A, colistin and OAA. For the analysis of microbiological failure, a bivariate logistic regression model was utilized employing the same methods as described above. Calculations were performed using SPSS v. 29. Approval was obtained from the institutional review boards of all participating centers; the need for informed consent was waived given the retrospective design.

## Results

A total of 360 patients with CPE bacteremia were identified: 226 with KPC, 109 NDM, 13 OXA48, 5 dual KPC/NDM, 3 VIM, 3 dual VIM/KPC and one dual KPC/OXA. A majority (71.4%) were from Italy; 72.2% were males and median age was 66 (IQR 55–75). *Klebsiella* spp. was the causative pathogen in 324 (90%) of episodes. Among 359 patients with data about antimicrobial therapy, 32.4% received appropriate empiric treatment while 87.7% received appropriate definitive treatment; 48.7% received combination therapy. Definitive therapy was colistin-based in 35.1% of patients, CAZ/AVI ± A in 28.2% and other in 23.4%; patients who received definitive therapy with CAZ/AVI ± A were almost exclusively (97.0%) from Italy. (Table S1). Overall 14-day mortality was 28.1%; 28-day mortality and microbiological failure were 35.9% and 18.1%, respectively.

**Fig. 1:**
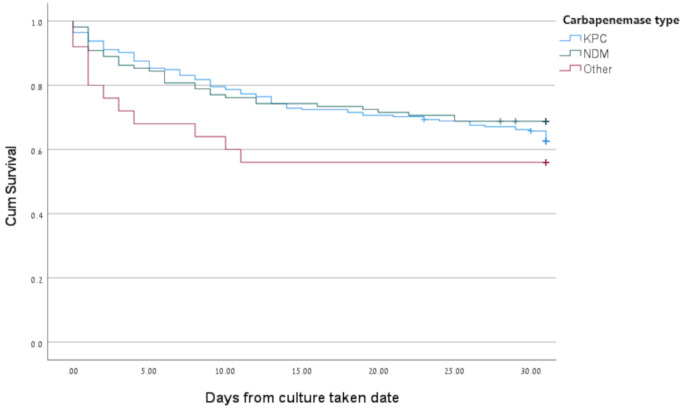
Carbapenamase type and cumulative mortality

Patients with NDM-producing isolates were older, had higher rates of malignancy, were more likely to be receiving steroid therapy, had higher Pitt bacteremia score at disease onset, had lower rates urinary tract infection (UTI) or biliary tract as the source of their infection, were less likely to receive combination therapy and more likely to receive definitive therapy with CAZ/AVI ± A as compared to those with KPC-producing isolates; other characteristics were similar (Table 1). In unadjusted analysis, no difference was observed in 14-day or 28-day mortality between the groups (Fig. 1); microbiological failure was higher in patients with NDM-producing isolates than those with KPC and other CPE (26.6% vs. 14.2% and 16.0%).

**Table 1:** Univariate analysis by carbapenemase type

	KPC *N* = 226	NDM *N* = 109	Other *N* = 25	*p* value	*p* value KPC&NDM
Country - Italy	162 (71.7%)	88 (80.7%)	7 (28.0%)	< 0.001	0.074
Age, median (IQR)	65 (54–73)	71 (60–77)	59 (50–81)	0.009	0.002
Male	161 (71.2%)	80 (73.4%)	19 (76.0%)	0.835	0.681
Functional status				0.254	0.169
Fully functional	165 (73.0%)	77 (70.6%)	15 (60.0%)		
Requires assistance	30 (13.3%)	22 (20.2%)	5 (20.0%)		
Bed-ridden	31 (13.7%)	10 (9.2%)	5 (20.0%)		
BMI, median (IQR) *N* = 133	25.5 (22.0-30.7)	25.0 (22.9–27.3)	26.9 (23.7–28.6)	0.398	0.685
Recent surgery	57 (25.2%)	35 (32.1%)	8 (32.0%)	0.372	0.186
Chronic kidney disease	53 (23.5%)	25 (22.9%)	6 (24.0%)	0.991	0.917
Diabetes mellitus	72 (31.9%)	40 (36.7%)	10 (40.0%)	0.544	0.379
Liver disease	28 (12.4%)	12 (11.0%)	5 (20.0%)	0.470	0.715
Ischemic heart disease	55 (24.3%)	29 (26.6%)	6 (24.0%)	0.898	0.653
Congestive heart failure	48 (21.2%)	21 (19.3%)	4 (16.0%)	0.786	0.676
Peripheral vascular disease	29 (12.8%)	9 (8.3%)	3 (12.0%)	0.464	0.216
Previous CVA	33 (14.6%)	14 (12.8%)	2 (8.0%)	0.634	0.664
Hemiplegia	13 (5.8%)	4 (3.7%)	2 (8.0%)	0.596	0.596
Dementia	22 (9.7%)	8 (7.3%)	2 (8.0%)	0.761	0.472
Peptic ulcer disease	15 (6.6%)	1 (0.9%)	2 (8.0%)	0.062	0.026
Connective tissue disease	11 (4.9%)	4 (3.7%)	1 (4.0%)	0.878	0.781
COPD	25 (11.1%)	20 (18.3%)	2 (8.0%)	0.132	0.067
Malignancy				0.021	0.027
Solid tumor, local	32 (14.2%)	26 (23.9%)	3 (12.0%)		
Solid tumor, metastases	16 (7.1%)	6 (5.5%)	0		
Hematologic	29 (12.8%)	5 (4.6%)	6 (24.0%)		
Organ transplant	24 (10.6%)	8 (7.3%)	4 (16.0%)	0.377	0.339
AIDS	2 (0.9%)	1 (0.9%)	0	0.893	1.000
Steroid therapy	55 (24.3%)	43 (39.4%)	10 (40.0%)	0.010	0.004
Other immunosuppressive medication	31 (13.7%)	12 (11.0%)	7 (28.0%)	0.085	0.488
Chemotherapy	33 (14.6%)	9 (8.3%)	6 (24.0%)	0.074	0.100
Charlson score, median (IQR)	5 (2–7)	5 (3–7)	4 (3–7)	0.932	0.712
Infection source				< 0.001	0.020
UTI or biliary tract	95 (42.0%)	29 (26.6%)	5 (20.0%)		
Pneumonia	15 (6.6%)	14 (12.%)	8 (32.0%)		
Skin and soft tissue	21 (9.3%)	9 (8.3%)	2 (8.0%)		
Other	95 (42.0%)	57 (52.3%)	10 (40.0%)		
Adequate source control	140 (61.9%)	71 (65.1%)	17 (68.0%)	0.750	0.571
Mechanical ventilation	74 (32.7%)	44 (40.4%)	9 (36.0%)	0.391	0.225
Vasopressors	68 (30.1%)	40 (36.7%)	12 (48.0%)	0.132	0.171
New onset dialysis	16 (7.1%)	10 (9.2%)	3 (12.0%)	0.607	0.502
Severe sepsis	85 (37.6%)	46 (42.2%)	14 (56.0%)	0.182	0.420
Pitt bacteremia score, median (IQR)	2 (1–4)	3 (2–6)	2 (1–8)	0.002	< 0.001
Neutrophils, median (IQR) *N* = 340	8342 (4500–14780)	10,674 (6961–15595)	7610 (1698–11620)	0.013	0.010
Platelets (thousands), median (IQR) *N* = 345	166 (72–262)	191 (104–267)	111 (24–173)	0.017	0.125
Hemoglobin (g/dL), median (IQR) *N* = 221	9.1 (8.3–10.4)	9.1 (8.4–10.4)	9.0 (8.4–9.6)	0.987	0.907
Sodium (mmol/L), median (IQR) *N* = 219	138 (135–141)	139 (136–142)	140 (137–146)	0.130	0.158
Creatinine (mg/dL), median (IQR) *N* = 285	1.16 (0.72–2.46)	1.20 (0.75–2.18)	1.34 (0.71–2.18)	0.878	0.688
Bilirubin (mg/dL), median (IQR) *N* = 305	1.0 (0.7-3.0)	0.9 (0.5–2.1)	1.3 (0.6–2.7)	0.028	0.010
Transaminases > 2XULN *N* = 348	34 (15.7%)	13 (12.1%)	4 (17.4%)	0.673	0.398
Albumin (g/dL), median (IQR) *N* = 200	2.7 (2.2–3.2)	2.8 (2.3–3.1)	2.7 (2.2–3.3)	0.971	0.831
Appropriate empiric treatment *N* = 359	60 (26.7%)	19 (17.4%)	7 (28.1%)	0.159	0.063
Appropriate definitive treatment *N* = 359	198 (88.0%)	97 (89.0%)	20 (80.0%)	0.457	0.791
Use of combination therapy *N* = 359	137 (60.9%)	25 (22.9%)	13 (52.0%)	< 0.001	< 0.001
Time from CTD to AAT (days), median (IQR) *N* = 310	1 (0–2)	1 (0–2)	2 (0–3)	0.701	0.658
Definitive therapy drug				< 0.001	< 0.001
Colistin	97 (49.2%)	18 (18.8%)	11 (57.9%)		
CAZ/AVI ± A	45 (22.8%)	57 (59.4%)	0		
Other	55 (27.9%)	21 (21.9%)	8 (42.1%)		
Alive 48 h *N* = 359	207 (92.0%)	98 (89.9%)	20 (80.0%)	0.146	0.524
Alive 7 days *N* = 359	189 (84.0%)	87 (79.8%)	17 (68.0%)	0.124	0.344
Alive 14 days *N* = 359	162 (72.0%)	82 (75.2%)	14 (56.0%)	0.155	0.533
Alive 28 days *N* = 359	141 (62.7%)	75 (68.8%)	14 (56.0%)	0.375	0.271
Microbiologic failure	32 (14.2%)	29 (26.6%)	4 (16.0%)	0.020	0.006

AAT– Appropriate antibiotic therapy; AIDS– Acquired immunodeficiency syndrome; BMI– Body mass index; CAZ/AVI ± A– Ceftazidime/Avibactam ± aztreonam; CI– Confidence interval; COPD– Chronic obstructive pulmonary disease; CTD– Culture taken date; CVA– Cerebrovascular accident; IQR– Interquartile range; KPC - *Klebsiella pneumoniae* carbapenemase; NDM– New Delhi metallo-β-lactamase; Ref– Reference; ULN– Upper limit of normal; UTI– Urinary tract infection

Among 334 patients with KPC- or NDM-producing isolates and data on antimicrobial therapy, 14-day mortality was 26.9%. In univariate analysis, mortality risk was associated with Israel as country of origin, increasing age, decreasing functional status, a number of chronic background conditions, nonsteroidal immunosuppressive medication, lack of adequate source control, markers of increased disease severity at CTD and definitive treatment with colistin-based therapy (Table 2). No difference was observed between patients who received definitive therapy with colistin alone (6/21) or as part of a combination (22/80, *p* = 0.922). In multivariable analysis, decreasing functional status, increasing Charlson score and Pitt bacteremia score and treatment with colistin as compared with CAZ/AVI ± A or OAA were predictors of mortality; no difference was observed between patients with KPC- and NDM-producing isolates (Table S2). To address the fact that almost all patients who received CAZ/AVI ± A were from Italy, we performed two additional sensitivity analyses: running the conditional logistic regression while stratifying by country and performing the analysis exclusively on patients from Italy. Neither affected the results apart from the fact that in the exclusively-Italian analysis, Pitt bacteremia score was not a significant predictor of mortality. The separate analyses by therapy type indicated similar results, with no observed association between carbapenemase type and 14-day mortality (Tables S3-5).

**Table 2:** Univariate predictors of 14-day mortality

	Deceased 14 days *N* = 90	Alive 14 days *N* = 244	*p* value - univariate
Country of origin - Italy	56 (62.2%)	193 (79.1%)	0.002
Age, median (IQR)	71 (60–79)	66 (54–73)	< 0.001
Male	60 (66.7%)	180 (73.8%)	0.200
Functional status			< 0.001
Fully functional	55 (61.1%)	186 (76.2%)	
Requires assistance	14 (15.6%)	38 (15.6%)	
Bed-ridden	21 (23.3%)	20 (8.2%)	
BMI, median (IQR) *N* = 118	25.0 (22.5–27.6)	25.2 (22.0–31.0)	0.604
Recent surgery	20 (22.2%)	72 (29.5%)	0.186
Chronic kidney disease	32 (35.6%)	46 (18.9%)	0.001
Diabetes mellitus	36 (40.0%)	75 (30.7%)	0.111
Liver disease	13 (14.4%)	26 (10.7%)	0.339
Ischemic heart disease	33 (36.7%)	51 (20.9%)	0.003
Congestive heart failure	33 (36.7%)	36 (14.8%)	< 0.001
Peripheral vascular disease	16 (17.8%)	22 (9.0%)	0.025
Previous CVA	23 (25.6%)	24 (9.8%)	< 0.001
Hemiplegia	5 (5.6%)	12 (4.9%)	0.784
Dementia	14 (15.6%)	16 (6.6%)	0.011
Peptic ulcer disease	7 (7.8%)	9 (3.7%)	0.121
Connective tissue disease	7 (7.8%)	8 (3.3%)	0.078
COPD	14 (15.6%)	31 (12.7%)	0.498
Malignancy			0.308
Solid tumor, local	10 (11.1%)	48 (19.7%)	
Solid tumor, metastases	6 (6.7%)	16 (6.6%)	
Hematologic	11 (12.2%)	23 (9.4%)	
Organ transplant	7 (7.8%)	25 (10.2%)	0.497
AIDS	2 (2.2%)	1 (0.4%)	0.178
Steroid therapy	21 (23.3%)	77 (31.6%)	0.143
Other immunosuppressive medication	7 (7.8%)	36 (14.8%)	0.091
Chemotherapy	10 (11.1%)	32 (13.1%)	0.624
Charlson score, median (IQR)	6 (4–8)	4 (2–6)	< 0.001
Infection source			0.119
UTI or biliary tract	30 (33.3%)	93 (38.1%)	
Pneumonia	13 (14.4%)	16 (6.6%)	
Skin and soft tissue	6 (6.7%)	24 (9.8%)	
Other	41 (45.6%)	111 (45.5%)	
Adequate source control	45 (50.0%)	165 (67.6%)	0.003
Mechanical ventilation	39 (43.3%)	79 (32.4%)	0.063
Vasopressors	47 (52.2%)	61 (25.0%)	< 0.001
New onset dialysis	15 (16.7%)	11 (4.5%)	< 0.001
Severe sepsis	55 (61.1%)	76 (31.1%)	< 0.001
Pitt bacteremia score, median (IQR)	4 (2–7)	2 (1–4)	< 0.001
Neutrophils, median (IQR) *N* = 315	10,069 (5500–15700)	8603 (5052–14633)	0.168
Platelets (thousands), median (IQR) *N* = 320	110 (58–200)	194 (92–271)	< 0.001
Hemoglobin (g/dL), median (IQR) *N* = 197	8.7 (8.1–10.2)	9.2 (8.4–10.4)	0.044
Sodium (mmol/L), median (IQR) *N* = 195	139 (136–142)	138 (135–141)	0.083
Creatinine (mg/dL), median (IQR) *N* = 260	1.97 (1.17–3.93)	1.00 (0.67–1.81)	< 0.001
Bilirubin (mg/dL), median (IQR) *N* = 282	2.0 (0.8–6.2)	1.0 (0.6–1.7)	< 0.001
Transaminases > 2XULN *N* = 323	13 (14.9%)	34 (14.4%)	0.904
Albumin (g/dL), median (IQR) *N* = 178	2.6 (1.9–3.1)	2.9 (2.3–3.2)	0.044
NDM	27 (30.0%)	82 (33.6%)	0.533
Appropriate empiric treatment *N* = 333	21 (23.3%)	58 (23.9%)	0.919
Appropriate definitive treatment *N* = 333	65 (72.2%)	230 (94.7%)	< 0.001
Use of combination therapy *N* = 333	41 (45.6%)	121 (49.8%)	0.492
Time from CTD to AAT (days), median (IQR) *N* = 290	1 (0–2)	0 (1–2)	0.810
1st Appropriate drug			< 0.001
Colistin	38 (42.2%)	77 (31.6%)	
CAZ/AVI ± A	11 (12.2%)	91 (37.3%)	
Other	17 (18.9%)	59 (24.2%)	
Definitive drug			< 0.001
Colistin	28 (31.1%)	73 (29.9%)	
No definitive therapy	46 (51.1%)	23 (9.4%)	
CAZ/AVI ± A	6 (6.7%)	94 (28.1%)	
Other	10 (11.1%)	54 (22.1%)	

AAT– Appropriate antibiotic therapy; AIDS– Acquired immunodeficiency syndrome; BMI– Body mass index; CAZ/AVI ± S– Ceftazidime/Avibactam ± aztreonam; CI– Confidence interval; COPD– Chronic obstructive pulmonary disease; CTD– Culture taken date; CVA– Cerebrovascular accident; IQR– Interquartile range; NDM– New Delhi metallo-β-lactamase; Ref– Reference; UTI– Urinary tract infection

At 14 days, patients treated with CAZ/AVI ± A had 82.8% less mortality risk than patients treated with colistin-based therapy. To address possible immortal time bias, we performed an analysis limited to patients who survived to day 7 and found that patients who received definitive treatment based on colistin had greater 14-day mortality (20/98) than those who received treatment based on CAZ/AVI ± A (4/99) or OAA (8/69, *p* = 0.002).

At 28 days, mortality was 35.3% with no difference observed between patients with KPC- and NDM-producing isolates (Table 3); the same result was obtained in multivariable analysis (Table S6). The overall rate of microbiological failure was 18.1%. In multivariable analysis, a significant difference in failure was not observed between patients with different CPE types although a trend remained (NDM vs. KPC, HR 2.745 95%CI 0.935–8.057) (Table 4).

**Table 3:** Univariate predictors of 28-day mortality

	Deceased 28 days *N* = 118	Alive 28 days *N* = 216	*p* value
Country of origin - Italy	78 (66.1%)	171 (79.2%)	0.009
Age, median (IQR)	70 (60–78)	66 (52–73)	< 0.001
Male	78 (66.1%)	162 (75.0%)	0.084
Functional status			0.007
Fully functional	75 (63.6%)	166 (76.9%)	
Requires assistance	20 (16.9%)	32 (14.8%)	
Bed-ridden	23 (19.5%)	18 (8.3%)	
BMI, median (IQR) *N* = 118	25.0 (22.2–28.1)	25.2 (22.2–31.0)	0.496
Recent surgery	30 (25.4%)	62 (28.7%)	0.521
Chronic kidney disease	39 (33.1%)	39 (18.1%)	0.002
Diabetes mellitus	48 (40.7%)	63 (29.2%)	0.033
Liver disease	18 (15.3%)	21 (9.7%)	0.132
Ischemic heart disease	42 (35.6%)	42 (19.4%)	0.001
Congestive heart failure	40 (33.9%)	29 (13.4%)	< 0.001
Peripheral vascular disease	18 (15.3%)	20 (9.3%)	0.099
Previous CVA	28 (23.7%)	19 (8.8%)	< 0.001
Hemiplegia	6 (5.1%)	11 (5.1%)	0.998
Dementia	15 (12.7%)	15 (6.9%)	0.078
Peptic ulcer disease	8 (6.8%)	8 (3.7%)	0.208
Connective tissue disease	7 (5.9%)	8 (3.7%)	0.347
COPD	18 (15.3%)	27 (12.5%)	0.481
Malignancy			0.417
Solid tumor, local	15 (12.7%)	43 (19.9%)	
Solid tumor, metastases	8 (6.8%)	14 (6.5%)	
Hematologic	12 (10.2%)	22 (10.2%)	
Organ transplant	11 (9.3%)	21 (9.7%)	0.905
AIDS	2 (1.7%)	1 (0.5%)	0.286
Steroid therapy	28 (23.7%)	70 (32.4%)	0.096
Other immunosuppressive medication	14 (11.9%)	29 (13.4%)	0.684
Chemotherapy	12 (10.2%)	30 (13.9%)	0.327
Charlson score, median (IQR)	6 (4–8)	4 (2–6)	< 0.001
Infection source			0.006
UTI or biliary tract	37 (31.4%)	86 (39.8%)	
Pneumonia	18 (15.3%)	11 (5.1%)	
Skin and soft tissue	7 (5.9%)	23 (10.6%)	
Other	56 (47.5%)	96 (44.4%)	
Adequate source control	62 (52.5%)	148 (68.5%)	0.004
Mechanical ventilation	55 (46.6%)	53 (24.5%)	< 0.001
Vasopressors	51 (43.2%)	67 (31.0%)	0.026
New onset dialysis	19 (16.1%)	7 (3.2%)	< 0.001
Severe sepsis	67 (56.8%)	64 (29.6%)	< 0.001
Pitt bacteremia score, median (IQR)	4 (2–6)	2 (1–4)	< 0.001
Neutrophils, median (IQR) *N* = 315	10,030 (5635–15840)	8455 (4768–14196)	0.085
Platelets (thousands), median (IQR) *N* = 320	115 (57–207)	203 (105–274)	< 0.001
Hemoglobin (g/dL), median (IQR) *N* = 197	8.9 (8.0-10.2)	9.2 (8.5–10.4)	0.049
Sodium (mmol/L), median (IQR) *N* = 195	139 (136–142)	137 (135–140)	0.040
Creatinine (mg/dL), median (IQR) *N* = 260	1.73 (1.01–3.62)	1.01 (0.67–1.81)	< 0.001
Bilirubin (mg/dL), median (IQR) *N* = 282	1.3 (0.7-4.0)	0.9 (0.6-2.0)	0.002
Transaminases > 2XULN *N* = 323	17 (14.9%)	30 (14.4%)	0.892
Albumin (g/dL), median (IQR) *N* = 178	2.5 (2.0-2.9)	2.9 (2.3–3.2)	0.001
NDM	34 (28.8%)	75 (34.7%)	0.271
Appropriate empiric treatment	31 (26.3%)	48 (22.3%)	0.418
Appropriate definitive treatment	91 (77.1%)	204 (94.9%)	< 0.001
Use of combination therapy	61 (51.7%)	101 (47.0%)	0.410
Time from CTD to AAT (days), median (IQR) *N* = 290	1 (0–3)	1 (0–2)	0.326
1st Appropriate drug			< 0.001
Colistin	51 (43.2%)	64 (29.6%)	
CAZ/AVI ± A	16 (13.6%)	86 (39.8%)	
Other	25 (21.2%)	51 (23.6%)	
Definitive drug			< 0.001
Colistin	40 (33.9%)	61 (28.2%)	
No definitive therapy	49 (41.5%)	20 (9.3%)	
CAZ/AVI ± A	11 (9.3%)	89 (41.2%)	
Other	18 (15.3%)	46 (21.3%)	

AAT– Appropriate antibiotic therapy; AIDS– Acquired immunodeficiency syndrome; BMI– Body mass index; CAZ/AVI ± A– Ceftazidime/Avibactam ± aztreonam; CI– Confidence interval; COPD– Chronic obstructive pulmonary disease; CTD– Culture taken date; CVA– Cerebrovascular accident; IQR– Interquartile range; NDM– New Delhi metallo-β-lactamase; Ref– Reference; ULN– Upper limit of normal; UTI– Urinary tract infection

**Table 4:** Predictors of Microbiological failure

	No failure *N* = 294	Microbiological failure *N* = 65	*p* value	OR (95%CI)– Multivariate analysis
Country of origin - Italy	211 (71.5%)	46 (70.8%)	0.903	
Age, median (IQR)	66 (55–74)	66 (56–77)	0.735	
Male	219 (74.2%)	41 (63.1%)	0.069	0.668 (0.238–1.872)
Functional status			0.541	
Fully functional	213 (72.2%)	44 (67.7%)		
Requires assistance	47 (15.9%)	10 (15.4%)		
Bed-ridden	35 (11.9%)	11 (16.9%)		
BMI, median (IQR) *N* = 133	25.0 (24.5–29.3)	26.0 (23.5–28.4)	0.499	
Recent surgery	83 (28.1%)	17 (26.2%)	0.747	
Chronic kidney disease	70 (23.7%)	14 (21.5%)	0.705	
Diabetes mellitus	99 (33.6%)	23 (35.4%)	0.778	
Liver disease	35 (11.9%)	10 (15.4%)	0.437	
Ischemic heart disease	72 (24.4%)	18 (27.7%)	0.580	
Congestive heart failure	56 (19.0%)	17 (26.2%)	0.193	
Peripheral vascular disease	38 (12.9%)	3 (4.6%)	0.082	
Previous CVA	36 (12.2%)	13 (20.0%)	0.097	0.571 (0.106–3.082)
Hemiplegia	15 (5.1%)	4 (6.2%)	0.759	
Dementia	26 (8.8%)	6 (9.2%)	0.915	
Peptic ulcer disease	16 (5.4%)	2 (3.1%)	0.752	
Connective tissue disease	13 (4.4%)	3 (4.6%)	1.000	
COPD	41 (13.9%)	6 (9.2%)	0.312	
Malignancy			0.613	
Solid tumor, local	53 (18.0%)	8 (12.3%)		
Solid tumor, metastases	19 (6.4%)	3 (4.6%)		
Hematologic	33 (11.2%)	7 (10.8%)		
Organ transplant	31 (10.5%)	5 (7.7%)	0.649	
AIDS	3 (1.0%)	0	1.000	
Steroid therapy	92 (31.2%)	16 (24.6%)	0.295	
Other immunosuppressive medication	44 (14.9%)	6 (9.2%)	0.230	
Chemotherapy	40 (13.6%)	8 (12.3%)	0.788	
Charlson score, median (IQR)	5 (3–7)	5 (3–7)	0.663	
Infection source			< 0.001	
UTI or biliary tract	113 (38.3%)	16 (24.6%)		Ref
Pneumonia	33 (11.2%)	4 (6.2%)		0.190 (0.019–1.863)
Skin and soft tissue	18 (6.1%)	14 (21.5%)		4.978 (1.243–19.933)
Other	131 (44.4%)	31 (47.7%)		1.034 (0.366–2.917)
Adequate source control	195 (66.1%)	33 (50.8%)	0.020	0.429 (0.165–1.115)
Mechanical ventilation	95 (32.2%)	32 (49.2%)	0.009	
Vasopressors	89 (30.2%)	31 (47.7%)	0.007	
New onset dialysis	17 (5.8%)	12 (18.5%)	< 0.001	
Severe sepsis	113 (38.3%)	32 (49.2%)	0.104	
Pitt bacteremia score, median (IQR)	2 (1–4)	4 (2–7)	0.001	1.082 (0.931–1.257)
Neutrophils, median (IQR) *N* = 340	8370 (4680–14442)	10,420 (7470–16840)	0.022	
Platelets (thousands), median (IQR) *N* = 345	175 (77–264)	141 (59–233)	0.152	
Hemoglobin (g/dL), median (IQR) *N* = 221	9.2 (8.3–10.4)	8.7 (8.2–9.2)	0.061	
Sodium (mmol/L), median (IQR) *N* = 219	138 (135–141)	140 (136–143)	0.070	
Creatinine (mg/dL), median (IQR) *N* = 285	1.12 (0.71–2.30)	1.44 (0.89–2.19)	0.450	
Bilirubin (mg/dL), median (IQR) *N* = 305	1.0 (0.6–2.4)	1.0 (0.6–2.9)	0.568	
Transaminases > 2XULN *N* = 348	42 (14.7%)	9 (14.3%)	0.927	
Albumin (g/dL), median (IQR) *N* = 200	2.8 (2.3–3.2)	2.3 (2.0-3.1)	0.035	0.489 (0.229–1.046)
CPE type			0.020	
KPC	194 (65.8%)	32 (49.2%)		Ref
NDM	80 (27.1%)	29 (44.6%)		2.745 (0.935–8.057)
Other	21 (7.1%)	4 (6.2%)		2.716 (0.651–11.339)
Appropriate empiric treatment	74 (25.2%)	12 (18.5%)	0.251	
Appropriate definitive treatment	261 (88.8%)	54 (83.1%)	0.205	
Use of combination therapy	141 (48.0%)	34 (52.3%)	0.526	
Time from CTD to AAT (days), median (IQR) *N* = 310	1 (0–2)	2 (0–3)	0.183	
1st Appropriate drug			0.368	
Colistin	104 (35.3%)	22 (33.8%)		
CAZ/AVI ± A	82 (27.8%)	20 (30.8%)		
Other	73 (24.7%)	11 (16.9%)		
Definitive drug			0.213	
Colistin	87 (29.5%)	22 (33.8%)		
CAZ/AVI ± A	82 (27.8%)	19 (29.2%)		
Other	65 (22.0%)	7 (10.8%)		

AAT– Appropriate antibiotic therapy; AIDS– Acquired immunodeficiency syndrome; BMI– Body mass index; CAZ/AVI ± A– Ceftazidime/Avibactam ± aztreonam; CI– Confidence interval; COPD– Chronic obstructive pulmonary disease; CPE– Carbapanemase-producing *Enterobacterales*; CTD– Culture taken date; CVA– Cerebrovascular accident; IQR– Interquartile range; KPC– *Klebsiella pneumoniae* carbapenemase; NDM– New Delhi metallo-β-lactamase; Ref– Reference; ULN– Upper limit of normal; UTI– Urinary tract infection

## Discussion

In a large, multicenter cohort study we did not observe an association between CPE type and mortality at 14 or 28 days in patients with BSI. Definitive antibiotic therapy with colistin was associated with a higher mortality rate than CAZ/AVI ± A.

The epidemiology of infections caused by CPE continues to change worldwide [12, 13]. Questions have arisen regarding potential differences between infections caused by differing carbapenemases including whether there are inherent differences in virulence between CPE types. In a previous study of a national cohort of CPE-carriers in Israel, higher rates of mortality were observed among patients colonized with KPC than with NDM [4]. That analysis was not intended to assess mortality as the primary outcome and was limited by a lack of clinical data resulting in numerous differences between the groups for which we could not correct. Until recently, the standard of care for these infections was colistin-based regimens. A major advance was made with the development of ceftazidime/avibactam which has been found in several studies to be superior to colistin [14–16]. The addition of aztreonam extends this option to infections caused by metallo-β-lactamases (MBL). However, even with the advent of more effective treatment, numerous patients continue to receive alternatives due to reasons of cost, availability and antimicrobial stewardship. For these reasons, cohort studies must take into account treatment regimens when analyzing outcomes in infections caused by differing carbapenemases.

Seo et al. performed a large hospital-based cohort of patients from the Republic of Korea with infections due to CPE [3]. In that study, most patients (648/859) were colonized with KPC and NDM at the outset; patients colonized by KPC were more likely to develop clinical infections and to die than those colonized by NDM. Among patients with infections initially or during the course of the study, those caused by KPC-producing isolates were found to have higher rates of mortality. Only 150 of the patients included had BSI and no data on the antibiotics used to treat these infections were provided or included in the analyses. In the recent ALARICO study, mortality was assessed in patients with BSI caused by carbapenem-resistant Gram-negative bacteria [10]. Of these, 304 and 77 were KPC- and MBL-producing isolates, respectively. A higher attributable mortality was identified among patients with MBL than KPC, however while data on the antibiotics used to treat these infections are provided in aggregate in the discussion, they were not included in the analyses. As noted in the study, half of the patients with KPC received CAZ/AVI while nearly two-thirds of patients with NDM received colistin, potentially skewing the results. In contrast, a study from Italy which analyzed outcomes in patients with BSI caused by CPE and which included antibiotic therapy in the analysis did not find a difference in mortality between KPC and MBL [11]. This study was limited by a small number of patients with MBL and by the fact that for half of the patients no data on antibiotic therapy were available.

Our study had a number of limitations. First, almost all of the patients who received CAZ/AVI were located in Italy and while we performed multiple variable analyses which took this fact into account, it may be impossible to completely adjust for associated confounders. Second, as with most previous studies of CPE we did not have data on bacterial clones. Although this was a multicenter study, it is possible that specific clones were associated with increased virulence and mortality. However, as this would tend to bias towards a difference in mortality between carbapenemase types we believe that data on clones would be unlikely to change the primary finding.

In conclusion, BSI caused by CPE continues to be associated with considerable mortality even with the introduction of more effective antibiotics. Previous findings of an association between carbapanemase type and increased mortality are likely due to antibiotic therapy and not inherent differences in virulence.

## Electronic supplementary material

Below is the link to the electronic supplementary material.

Supplementary Material 1

Supplementary Material 2

Supplementary Material 3

Supplementary Material 4

Supplementary Material 5

Supplementary Material 6

## Data Availability

No datasets were generated or analysed during the current study.
