# Supplementary material for: Carbapenemase type and mortality in blood-stream infections caused by carbapenemase-producing enterobacterales: a multicenter retrospective cohort study
Source: Infection. 2025 Jun 16;53(6):2491–501. doi: 10.1007/s15010-025-02584-y (PMC12675559; doi:10.1007/s15010-025-02584-y)
Supplement: Supplementary file 1 — Supplementary Material 1 [file 15010_2025_2584_MOESM1_ESM.docx]

**Table S1: Univariate analysis by definitive treatment type**

|  | Colistin  N=101 | CAZ/AVI ± Aztreonam N=100 | Other  N=64 | *p* value |
| --- | --- | --- | --- | --- |
| Country - Italy | 67 (66.3%) | 97 (97.0%) | 73 (67.2%) | <0.001 |
| Age, median (IQR) | 65 (54-73) | 68 (60-74) | 65 (55-74) | 0.332 |
| Male | 64 (63.4%) | 72 (72.0%) | 47 (73.4%) | 0.285 |
| Functional status |  |  |  | 0.526 |
| Fully functional | 75 (74.3%) | 79 (79.0%) | 45 (70.3%) |  |
| Requires assistance | 14 (13.9%) | 14 (14.0%) | 9 (14.1%) |  |
| Bed-ridden | 12 (11.9%) | 7 (7.0%) | 10 (15.6%) |  |
| BMI, median (IQR) N=96 | 25.4 (23.5-32.9) | 25.0 (21.8-27.2) | 25.5 (20.8-29.5) | 0.236 |
| Recent surgery | 26 (25.7%) | 31 (31.0%) | 77 (29.1%) | 0.647 |
| Chronic kidney disease | 19 (18.8%) | 23 (23.0%) | 15 (23.4%) | 0.702 |
| Diabetes mellitus | 22 (21.8%) | 36 (36.0%) | 19 (29.7%) | 0.084 |
| Liver disease | 12 (11.9%) | 11 (11.0%) | 5 (7.8%) | 0.698 |
| Ischemic heart disease | 22 (21.8%) | 21 (21.0%) | 17 (26.6%) | 0.684 |
| Congestive heart failure | 20 (19.8%) | 14 (14.0%) | 15 (23.4%) | 0.288 |
| Peripheral vascular disease | 11 (10.9%) | 8 (8.0%) | 4 (6.3%) | 0.560 |
| Previous CVA | 11 (10.9%) | 7 (7.0%) | 9 (14.1%) | 0.330 |
| Hemiplegia | 6 (5.9%) | 3 (3.0%) | 3 (4.7%) | 0.604 |
| Dementia | 8 (7.9%) | 6 (6.0%) | 9 (14.1%) | 0.190 |
| Peptic ulcer disease | 8 (7.9%) | 1 (1.0%) | 2 (3.1%) | 0.043 |
| Connective tissue disease | 4 (4.0%) | 4 (4.0%) | 5 (7.8%) | 0.466 |
| COPD | 12 (11.9%) | 9 (9.0%) | 8 (12.5%) | 0.727 |
| Malignancy |  |  |  | 0.044 |
| Solid tumor, local | 18 (17.8%) | 21 (21.0%) | 13 (20.3%) |  |
| Solid tumor, metastases | 6 (5.9%) | 7 (7.0%) | 6 (9.4%) |  |
| Hematologic | 16 (15.8%) | 2 (2.0%) | 9 (14.1%) |  |
| Organ transplant | 8 (7.9%) | 13 (13.0%) | 5 (7.8%) | 0.397 |
| AIDS | 2 (2.0%) | 1 (1.0%) | 0 | 0.497 |
| Steroid therapy | 29 (28.7%) | 37 (37.0%) | 17 (26.6%) | 0.288 |
| Other immunosuppressive medication | 12 (11.9%) | 13 (13.0%) | 11 (17.2%) | 0.611 |
| Chemotherapy | 15 (14.9%) | 8 (8.0%) | 13 (20.3%) | 0.072 |
| Charlson score, median (IQR) | 5 (2-7) | 4 (3-6) | 5 (3-8) | 0.539 |
| Infection source |  |  |  | 0.685 |
| UTI or biliary tract | 44 (43.6%) | 33 (33.0%) | 28 (43.8%) |  |
| Pneumonia | 5 (5.0%) | 8 (8.0%) | 5 (7.8%) |  |
| Skin and soft tissue | 10 (9.9%) | 9 (9.0%) | 6 (9.4%) |  |
| Other | 42 (41.6%) | 50 (50.0%) | 25 (39.1%) |  |
| Adequate source control | 71 (70.3%) | 72 (72.0%) | 36 (56.3%) | 0.083 |
| Mechanical ventilation | 36 (35.6%) | 36 (36.0%) | 12 (18.8%) | 0.038 |
| Vasopressors | 26 (25.7%) | 28 (28.0%) | 19 (29.7%) | 0.851 |
| New onset dialysis | 4 (4.0%) | 7 (7.0%) | 3 (4.7%) | 0.610 |
| Severe sepsis | 40 (39.6%) | 35 (35.0%) | 17 (26.6%) | 0.229 |
| Pitt bacteremia score, median (IQR) | 2 (1-4) | 3 (1-5) | 2 (1-4) | 0.820 |
| Time from CTD to AAT (days), median (IQR) N=258 | 1 (0-2) | 1 (0-2) | 2 (0-3) | 0.122 |
| KPC | 87 (86.1%) | 42 (42.0%) | 48 (75.0%) | <0.001 |

AAT – Appropriate antibiotic therapy; AIDS – Acquired immunodeficiency syndrome; BMI – Body mass index; CAZ/AVI – Ceftazidime/Avibactam; CI – Confidence interval; COPD – Chronic obstructive pulmonary disease; CTD – Culture taken date; CVA – Cerebrovascular accident; IQR – Interquartile range; KPC - *Klebsiella pneumoniae* carbapenemase; Ref – Reference; UTI – Urinary tract infection
