# Supplementary material for: Carbapenemase type and mortality in blood-stream infections caused by carbapenemase-producing enterobacterales: a multicenter retrospective cohort study
Source: Infection. 2025 Jun 16;53(6):2491–501. doi: 10.1007/s15010-025-02584-y (PMC12675559; doi:10.1007/s15010-025-02584-y)
Supplement: Supplementary file 2 — Supplementary Material 2 [file 15010_2025_2584_MOESM2_ESM.docx]

**Table S2: Multiple variable analysis of 14-day mortality**

| Variable | HR | Lower 95% CI | Upper 95% CI | *p* value |
| --- | --- | --- | --- | --- |
| Age | 1.023 | 0.996 | 1.051 | 0.089 |
| Functional status – Independent |  |  |  | Ref |
| Functional status – Requires assistance | 0.352 | 0.102 | 1.214 | 0.098 |
| Functional status - Bedridden | 2.887 | 1.448 | 5.755 | 0.003 |
| Charlson score | 1.152 | 1.023 | 1.297 | 0.019 |
| Adequate source control | 0.681 | 0.360 | 1.290 | 0.239 |
| Pitt bacteremia score | 1.166 | 1.033 | 1.316 | 0.013 |
| NDM | 1.109 | 0.475 | 2.587 | 0.811 |
| Colistin |  |  |  | Ref |
| CAZ/AVI±A | 0.172 | 0.063 | 0.473 | <0.001 |
| Other | 0.378 | 0.179 | 0.800 | 0.011 |

CAZ/AVI±A – Ceftazidime/Avibactam ± aztreonam; CI – Confidence interval; HR – Hazard ratio; NDM – New Delhi metallo-β-lactamase; Ref – Reference
