# Supplementary material for: Carbapenemase type and mortality in blood-stream infections caused by carbapenemase-producing enterobacterales: a multicenter retrospective cohort study
Source: Infection. 2025 Jun 16;53(6):2491–501. doi: 10.1007/s15010-025-02584-y (PMC12675559; doi:10.1007/s15010-025-02584-y)
Supplement: Supplementary file 3 — Supplementary Material 3 [file 15010_2025_2584_MOESM3_ESM.docx]

**Table S3: Predictors of 14-day mortality in patients receiving colistin as definitive therapy**

|  | Deceased 14 days  N=28 | Alive 14 days  N=73 | *p* value |
| --- | --- | --- | --- |
| Country of origin - Italy | 19 (67.9%) | 48 (65.8%) | 0.841 |
| Age, median (IQR) | 64 (58-73) | 65 (46-73) | 0.268 |
| Male | 16 (57.1%) | 48 (65.8%) | 0.421 |
| Functional status |  |  | 0.004 |
| Fully functional | 18 (64.3%) | 57 (78.1%) |  |
| Requires assistance | 2 (7.1%) | 12 (16.4%) |  |
| Bed-ridden | 8 (28.6%) | 4 (5.5%) |  |
| BMI, median (IQR) N=36 | 25.3 (23.5-27.8) | 25.6 (23.5-38.5) | 0.311 |
| Recent surgery | 5 (17.9%) | 21 (28.8%) | 0.262 |
| Chronic kidney disease | 8 (28.6%) | 11 (15.1%) | 0.120 |
| Diabetes mellitus | 6 (21.4%) | 16 (21.9%) | 0.957 |
| Liver disease | 4 (14.3%) | 8 (11.0%) | 0.733 |
| Ischemic heart disease | 10 (35.7%) | 12 (16.4%) | 0.036 |
| Congestive heart failure | 9 (32.1%) | 11 (15.1%) | 0.054 |
| Peripheral vascular disease | 4 (14.3%) | 7 (9.6%) | 0.492 |
| Previous CVA | 7 (25.0%) | 4 (5.5%) | 0.009 |
| Hemiplegia | 1 (3.6%) | 5 (6.8%) | 1.000 |
| Dementia | 4 (14.3%) | 4 (5.5%) | 0.212 |
| Peptic ulcer disease | 3 (10.7%) | 5 (6.8%) | 0.682 |
| Connective tissue disease | 3 (10.7%) | 1 (1.4%) | 0.064 |
| COPD | 4 (14.3%) | 8 (11.0%) | 0.733 |
| Malignancy |  |  | 0.127 |
| Solid tumor, local | 3 (10.7%) | 15 (20.5%) |  |
| Solid tumor, metastases | 4 (14.3%) | 2 (2.7%) |  |
| Hematologic | 4 (14.3%) | 12 (16.4%) |  |
| Organ transplant | 2 (7.1%) | 6 (8.2%) | 1.000 |
| AIDS | 2 (7.1%) | 0 | 0.075 |
| Steroid therapy | 5 (17.9%) | 24 (32.9%) | 0.135 |
| Other immunosuppressive medication | 2 (7.1%) | 10 (13.7%) | 0.502 |
| Chemotherapy | 4 (14.3%) | 11 (15.1%) | 1.000 |
| Charlson score, median (IQR) | 6 (3-9) | 4 (2-6) | 0.001 |
| Infection source |  |  | 0.317 |
| UTI or biliary tract | 13 (46.4%) | 31 (42.5%) |  |
| Pneumonia | 3 (10.7%) | 2 (2.7%) |  |
| Skin and soft tissue | 3 (10.7%) | 7 (9.6%) |  |
| Other | 9 (32.1%) | 33 (45.2%) |  |
| Adequate source control | 17 (60.7%) | 54 (74.0%) | 0.192 |
| Mechanical ventilation | 11 (39.3%) | 25 (34.2%) | 0.636 |
| Vasopressors | 9 (32.1%) | 17 (23.3%) | 0.362 |
| New onset dialysis | 2 (7.1%) | 2 (2.7%) | 0.307 |
| Severe sepsis | 15 (53.6%) | 25 (34.2%) | 0.075 |
| Pitt bacteremia score, median (IQR) | 3 (1-6) | 2 (1-4) | 0.215 |
| Neutrophils, median (IQR) N=96 | 9600 (5765-19051) | 8380 (3960-15283) | 0.184 |
| Platelets (thousands), median (IQR) N=98 | 95 (45-126) | 186 (77-262) | 0.009 |
| Hemoglobin (g/dL), median (IQR) N=58 | 8.8 (8.3-9.6) | 9.1 (7.8-11.0) | 0.422 |
| Sodium (mmol/L), median (IQR) N=57 | 139 (133-142) | 138 (136-141) | 0.932 |
| Creatinine (mg/dL), median (IQR) N=64 | 1.37 (0.87-2.69) | 0.85 (0.61-1.32) | 0.017 |
| Bilirubin (mg/dL), median (IQR) N=85 | 2.1 (1.0-5.0) | 1.0 (0.6-1.7) | 0.006 |
| Transaminases >2XULN N=97 | 5 (20.0%) | 10 (13.9%) | 0.467 |
| Albumin (g/dL), median (IQR) N=53 | 2.2 (1.9-2.7) | 2.8 (2.3-3.1) | 0.001 |
| NDM | 2 (7.1%) | 12 (16.4%) | 0.339 |
| Appropriate empiric treatment | 8 (28.6%) | 23 (31.5%) | 0.775 |
| Use of combination therapy | 22 (78.6%) | 58 (79.5%) | 0.922 |
| Time from CTD to AAT (days), median (IQR) | 1 (0-3) | 1 (0-2) | 0.778 |

AAT – Appropriate antibiotic therapy; AIDS – Acquired immunodeficiency syndrome; BMI – Body mass index; CI – Confidence interval; COPD – Chronic obstructive pulmonary disease; CTD – Culture taken date; CVA – Cerebrovascular accident; IQR – Interquartile range; NDM – New Delhi metallo-β-lactamase; Ref – Reference; ULN – Upper limit of normal; UTI – Urinary tract infection
