# Supplementary material for: Carbapenemase type and mortality in blood-stream infections caused by carbapenemase-producing enterobacterales: a multicenter retrospective cohort study
Source: Infection. 2025 Jun 16;53(6):2491–501. doi: 10.1007/s15010-025-02584-y (PMC12675559; doi:10.1007/s15010-025-02584-y)
Supplement: Supplementary file 4 — Supplementary Material 4 [file 15010_2025_2584_MOESM4_ESM.docx]

**Table S4: Predictors of 14-day mortality in patients receiving ceftazidime/avibactam ± aztreonam as definitive therapy**

|  | Deceased 14 days  N=6 | Alive 14 days  N=94 | *p* value |
| --- | --- | --- | --- |
| Country of origin - Italy | 6 (100%) | 91 (96.8%) | 0.657 |
| Age, median (IQR) | 82 (77-89) | 68 (58-73) | <0.001 |
| Male | 3 (50.0%) | 69 (73.4%) | 0.345 |
| Functional status |  |  | <0.001 |
| Fully functional | 3 (50.0%) | 76 (80.9%) |  |
| Requires assistance | 0 | 14 (14.9%) |  |
| Bed-ridden | 3 (50.0%) | 4 (4.3%) |  |
| BMI, median (IQR) N=38 | 20.0 (18.0-*) | 25.0 (22.0-27.7) | 0.247 |
| Recent surgery | 1 (16.7%) | 30 (31.9%) | 0.663 |
| Chronic kidney disease | 4 (66.7%) | 19 (20.2%) | 0.024 |
| Diabetes mellitus | 2 (33.3%) | 34 (36.2%) | 1.000 |
| Liver disease | 0 | 11 (11.7%) | 1.000 |
| Ischemic heart disease | 2 (33.3%) | 19 (20.2%) | 0.603 |
| Congestive heart failure | 3 (50.0%) | 11 (11.7%) | 0.034 |
| Peripheral vascular disease | 0 | 8 (8.5%) | 1.000 |
| Previous CVA | 2 (33.3%) | 5 (5.3%) | 0.055 |
| Hemiplegia | 1 (16.7%) | 2 (2.1%) | 0.171 |
| Dementia | 1 (16.7%) | 5 (5.3%) | 0.317 |
| Peptic ulcer disease | 0 | 1 (1.1%) | 1.000 |
| Connective tissue disease | 1 (16.7%) | 3 (3.2%) | 0.222 |
| COPD | 0 | 9 (9.6%) | 1.000 |
| Malignancy |  |  | 0.434 |
| Solid tumor, local | 0 | 21 (22.3%) |  |
| Solid tumor, metastases | 0 | 7 (7.4%) |  |
| Hematologic | 0 | 2 (2.1%) |  |
| Organ transplant | 1 (16.7%) | 12 (12.8%) | 0.576 |
| AIDS | 0 | 1 (1.1%) | 1.000 |
| Steroid therapy | 3 (50.0%) | 34 (36.2%) | 0.667 |
| Other immunosuppressive medication | 0 | 13 (13.8%) | 1.000 |
| Chemotherapy | 0 | 8 (8.5%) | 1.000 |
| Charlson score, median (IQR) | 6 (5-7) | 4 (3-6) | 0.049 |
| Infection source |  |  | 0.649 |
| UTI or biliary tract | 3 (50.0%) | 30 (31.9%) |  |
| Pneumonia | 0 | 8 (8.5%) |  |
| Skin and soft tissue | 0 | 9 (9.6%) |  |
| Other | 3 (50.0%) | 47 (50.0%) |  |
| Adequate source control | 2 (33.3%) | 70 (74.5%) | 0.050 |
| Mechanical ventilation | 1 (16.7%) | 35 (37.2%) | 0.415 |
| Vasopressors | 3 (50.0%) | 25 (26.6%) | 0.345 |
| New onset dialysis | 2 (33.3%) | 5 (5.3%) | 0.055 |
| Severe sepsis | 3 (50.0%) | 32 (34.0%) | 0.420 |
| Pitt bacteremia score, median (IQR) | 4 (2-6) | 2 (1-5) | 0.339 |
| Neutrophils, median (IQR) N=93 | 14968 (9765-22310) | 8800 (5584-14100) | 0.061 |
| Platelets (thousands), median (IQR) N=94 | 157 (80-218) | 209 (117-274) | 0.176 |
| Hemoglobin (g/dL), median (IQR) N=49 | 9.1 (9.1-9.1) | 9.3 (8.6-10.4) | 0.857 |
| Sodium (mmol/L), median (IQR) N=47 | 132 (132-132) | 138 (135-140) | 0.213 |
| Creatinine (mg/dL), median (IQR) N=85 | 2.18 (1.03-3.48) | 0.90 (0.61-1.58) | 0.046 |
| Bilirubin (mg/dL), median (IQR) N=85 | 0.4 (0.2-1.3) | 0.9 (0.6-1.5) | 0.077 |
| Transaminases >2XULN N=96 | 0 | 15 (16.7%) | 0.585 |
| Albumin (g/dL), median (IQR) N=49 | 2.7 (2.6-*) | 2.7 (2.2-3.0) | 0.939 |
| NDM | 5 (83.3%) | 53 (56.4%) | 0.396 |
| Appropriate empiric treatment | 3 (50.0%) | 13 (13.8%) | 0.050 |
| Use of combination therapy | 2 (33.3%) | 29 (30.9%) | 1.000 |
| Time from CTD to AAT (days), median (IQR) N=94 | 2 (0-4) | 1 (0-2) | 0.334 |

AAT – Appropriate antibiotic therapy; AIDS – Acquired immunodeficiency syndrome; BMI – Body mass index; CI – Confidence interval; COPD – Chronic obstructive pulmonary disease; CTD – Culture taken date; CVA – Cerebrovascular accident; IQR – Interquartile range; NDM – New Delhi metallo-β-lactamase; Ref – Reference; ULN – Upper limit of normal; UTI – Urinary tract infection
