# Supplementary material for: Carbapenemase type and mortality in blood-stream infections caused by carbapenemase-producing enterobacterales: a multicenter retrospective cohort study
Source: Infection. 2025 Jun 16;53(6):2491–501. doi: 10.1007/s15010-025-02584-y (PMC12675559; doi:10.1007/s15010-025-02584-y)
Supplement: Supplementary file 5 — Supplementary Material 5 [file 15010_2025_2584_MOESM5_ESM.docx]

**Table S5: Predictors of 14-day mortality in patients receiving other appropriate antibiotics as definitive therapy**

|  | Deceased 14 days  N=10 | Alive 14 days  N=54 | *p* value |
| --- | --- | --- | --- |
| Country of origin - Italy | 4 (40.0%) | 39 (72.2%) | 0.068 |
| Age, median (IQR) | 73 (67-79) | 64 (54-72) | 0.015 |
| Male | 6 (60.0%) | 41 (75.9%) | 0.295 |
| Functional status |  |  | 0.069 |
| Fully functional | 5 (50.0%) | 40 (74.1%) |  |
| Requires assistance | 1 (10.0%) | 8 (14.8%) |  |
| Bed-ridden | 4 (40.0%) | 6 (11.1%) |  |
| BMI, median (IQR) N=22 | 25.0 (22.3-*) | 26.0 (20.6-29.8) | 0.651 |
| Recent surgery | 2 (20.0%) | 18 (33.3%) | 0.486 |
| Chronic kidney disease | 4 (40.0%) | 11 (20.4%) | 0.226 |
| Diabetes mellitus | 3 (30.0%) | 16 (29.6%) | 1.000 |
| Liver disease | 0 | 5 (9.3%) | 1.000 |
| Ischemic heart disease | 4 (40.0%) | 13 (24.1%) | 0.435 |
| Congestive heart failure | 5 (50.0%) | 10 (18.5%) | 0.031 |
| Peripheral vascular disease | 1 (10.0%) | 3 (5.6%) | 0.502 |
| Previous CVA | 0 | 9 (16.7%) | 0.333 |
| Hemiplegia | 0 | 3 (5.6%) | 1.000 |
| Dementia | 2 (20.0%) | 7 (13.0%) | 0.622 |
| Peptic ulcer disease | 1 (10.0%) | 1 (1.9%) | 0.290 |
| Connective tissue disease | 1 (10.0%) | 4 (7.4%) | 1.000 |
| COPD | 1 (10.0%) | 7 (13.0%) | 1.000 |
| Malignancy |  |  | 0.697 |
| Solid tumor, local | 2 (20.0%) | 11 (20.4%) |  |
| Solid tumor, metastases | 0 | 6 (11.1%) |  |
| Hematologic | 2 (20.0%) | 7 (13.0%) |  |
| Organ transplant | 1 (10.0%) | 4 (7.4%) | 1.000 |
| AIDS | 0 | 0 |  |
| Steroid therapy | 4 (40.0%) | 13 (24.1%) | 0.435 |
| Other immunosuppressive medication | 2 (20.0%) | 9 (16.7%) | 1.000 |
| Chemotherapy | 1 (10.0%) | 12 (22.2%) | 0.672 |
| Charlson score, median (IQR) | 5 (4-8) | 5 (3-7) | 0.402 |
| Infection source |  |  | 0.663 |
| UTI or biliary tract | 4 (40.0%) | 24 (44.4%) |  |
| Pneumonia | 1 (10.0%) | 4 (7.4%) |  |
| Skin and soft tissue | 0 | 6 (11.1%) |  |
| Other | 5 (50.0%) | 20 (37.0%) |  |
| Adequate source control | 8 (80.0%) | 28 (51.9%) | 0.099 |
| Mechanical ventilation | 3 (30.0%) | 9 (16.7%) | 0.381 |
| Vasopressors | 8 (80.0%) | 11 (20.4%) | <0.001 |
| New onset dialysis | 2 (20.0%) | 1 (1.9%) | 0.061 |
| Severe sepsis | 6 (60.0%) | 11 (20.4%) | 0.009 |
| Pitt bacteremia score, median (IQR) | 4 (3-6) | 2 (1-3) | 0.003 |
| Neutrophils, median (IQR) N=63 | 14940 (4127-21146) | 8570 (6330-15620) | 0.288 |
| Platelets (thousands), median (IQR) | 189 (64-235) | 205 (104-309) | 0.411 |
| Hemoglobin (g/dL), median (IQR) N=49 | 9.3 (8.3-10.6) | 9.3 (8.7-10.7) | 0.623 |
| Sodium (mmol/L), median (IQR) N=49 | 136 (131-139) | 136 (134-140) | 0.330 |
| Creatinine (mg/dL), median (IQR) N=59 | 2.29 (1.30-4.68) | 1.11 (0.75-1.81) | 0.035 |
| Bilirubin (mg/dL), median (IQR) N=57 | 1.4 (0.6-2.7) | 0.9 (0.4-1.3) | 0.280 |
| Transaminases >2XULN N=63 | 0 | 5 (9.4%) | 0.311 |
| Albumin (g/dL), median (IQR) N=42 | 2.8 (2.6-3.1) | 3.0 (2.4-3.3) | 0.713 |
| NDM | 3 (30.0%) | 13 (24.1%) | 0.701 |
| Appropriate empiric treatment | 5 (50.0%) | 19 (35.2%) | 0.374 |
| Use of combination therapy | 5 (50.0%) | 29 (53.7%) | 0.829 |
| Time from CTD to AAT (days), median (IQR) N=63 | 1 (0-2) | 2 (0-3) | 0.407 |

AAT – Appropriate antibiotic therapy; AIDS – Acquired immunodeficiency syndrome; BMI – Body mass index; CI – Confidence interval; COPD – Chronic obstructive pulmonary disease; CTD – Culture taken date; CVA – Cerebrovascular accident; IQR – Interquartile range; NDM – New Delhi metallo-β-lactamase; Ref – Reference; ULN – Upper limit of normal; UTI – Urinary tract infection
