# Supplementary material for: Carbapenemase type and mortality in blood-stream infections caused by carbapenemase-producing enterobacterales: a multicenter retrospective cohort study
Source: Infection. 2025 Jun 16;53(6):2491–501. doi: 10.1007/s15010-025-02584-y (PMC12675559; doi:10.1007/s15010-025-02584-y)
Supplement: Supplementary file 6 — Supplementary Material 6 [file 15010_2025_2584_MOESM6_ESM.docx]

**Table S6: Multiple variable analysis of 28-day mortality**

| Variable | HR | Lower 95% CI | Upper 95% CI | *p* value |
| --- | --- | --- | --- | --- |
| Age | 1.021 | 0.999 | 1.043 | 0.058 |
| Functional status – Independent |  |  |  | Ref |
| Functional status – Requires assistance | 0.699 | 0.331 | 1.476 | 0.348 |
| Functional status - Bedridden | 1.960 | 1.057 | 3.632 | 0.033 |
| Charlson score | 1.118 | 1.014 | 1.231 | 0.025 |
| Adequate source control | 0.735 | 0.444 | 1.217 | 0.232 |
| Pitt bacteremia score | 1.167 | 1.061 | 1.284 | 0.001 |
| NDM | 1.012 | 0.534 | 1.921 | 0.970 |
| Colistin |  |  |  | Ref |
| CAZ/AVI±A | 0.207 | 0.098 | 0.439 | <0.001 |
| Other | 0.517 | 0.290 | 0.922 | 0.025 |

CAZ/AVI±A – Ceftazidime/Avibactam ± aztreonam; CI – Confidence interval; HR – Hazard ratio; NDM – New Delhi metallo-β-lactamase; Ref – Reference
